# Exploring immuno-regulatory mechanisms in the tumor microenvironment: Model and design of protocols for cancer remission

**DOI:** 10.1371/journal.pone.0203030

**Published:** 2018-09-05

**Authors:** Piyali Ganguli, Ram Rup Sarkar

**Affiliations:** 1 Chemical Engineering and Process Development Division, CSIR-National Chemical Laboratory, Pune, Maharashtra, India; 2 Academy of Scientific & Innovative Research (AcSIR), CSIR-NCL Campus, Pune, India; Università degli Studi della Campania, ITALY

## Abstract

The tumor microenvironment comprising of the immune cells and cytokines acts as the ‘soil’ that nourishes a developing tumor. Lack of a comprehensive study of the interactions of this tumor microenvironment with the heterogeneous sub-population of tumor cells that arise from the differentiation of Cancer Stem Cells (CSC), i.e. the ‘seed’, has limited our understanding of the development of drug resistance and treatment failures in Cancer. Based on this seed and soil hypothesis, for the very first time, we have captured the concept of CSC differentiation and tumor-immune interaction into a generic model that has been validated with known experimental data. Using this model we report that as the CSC differentiation shifts from symmetric to asymmetric pattern, resistant cancer cells start accumulating in the tumor that makes it refractory to therapeutic interventions. Model analyses unveiled the presence of feedback loops that establish the dual role of M2 macrophages in regulating tumor proliferation. The study further revealed oscillations in the tumor sub-populations in the presence of T_H1_ derived IFN-γ that eliminates CSC; and the role of IL10 feedback in the regulation of T_H1_/T_H2_ ratio. These analyses expose important observations that are indicative of Cancer prognosis. Further, the model has been used for testing known treatment protocols to explore the reasons of failure of conventional treatment strategies and propose an improvised protocol that shows promising results in suppressing the proliferation of all the cellular sub-populations of the tumor and restoring a healthy T_H1_/T_H2_ ratio that assures better Cancer remission.

## 1. Introduction

A malignant tumor is formed of heterogeneous population of cells. According to Cancer Stem Cell (CSC) Hypothesis, this tumor of heterogeneous cells is formed from a distinct group of cells having stem-like properties that are able to differentiate and renew for an indefinite period of time [1]. Popularly referred to as the Seed and Soil hypothesis, researchers believe that the CSCs acts like ‘seed’ and form the tumor initiating population of cells, that is responsible for the growth, sustenance, metastasis and relapse of Cancer [2]. These CSCs have the ability to differentiate both symmetrically and asymmetrically to form the terminally differentiated cancer cells as well as renew the pool of CSCs [3]. However, during proliferation, various extrinsic and intrinsic environmental factors give rise to random mutational events, such as, chromosomal breakage, translocation, aberrant signalling events and drug efflux, which are responsible for transformation and adaptation of the cell to resist the effect of drug and conventional therapeutic strategies [4]. This results in the formation of distinct cellular sub-populations that are drug resistant and impair the treatment of cancer.

On the other hand, the tumor microenvironment, composed mainly of the immune cells and the cytokines, plays a crucial role in determining cancer prognosis [5]. As the tumor develops, each of the tumor cell sub-populations starts manipulating the microenvironment and induces the production of pro-tumorigenic molecules. The CSCs and the Cancer cells induce the production of immune-modulatory molecules such as IL-10, IL-13 and TGF-β that are conducive to the proliferation of the M2-Tumor Associated Macrophages (M2-TAM), the Type II T-helper (T_H2_) cells and the T-regulatory (Treg) cells [6, 7]. The IL-10 mediated positive feedback loop between the tumor and the M2-TAMs helps in the rapid proliferation of the tumor sub-populations and the progression of the disease [8]. The CSCs also expresses high levels of co-inhibitory molecule PD-L1 that inhibit the activation of Cytotoxic T (Tc) cells [9]. Additionally, the CSC also tries to evade recognition by the immune cell by suppressing the expression of Major Histocompatibility Complex (MHC) by the macrophage cells in the tumor microenvironment. This is achieved by the release of exosomal miRNAs, such as miR-9 and miR-21, into the microenvironment by the tumor that are taken up by the immune cells, mediating changes in the cytokine expression pattern, antigen-recognition and immune responses [10, 11]. Along with these strategies of immune evasion, CSC also secretes VEGF, a growth factor that promotes angiogenesis during tumor progression and plays a pivotal role in suppressing the maturation of the T cells [12, 13]. These chemokines, cytokines and growth factors secreted by the stem cells lead the system to an inflammatory state. This also mediates a crosstalk between different groups of cells in the tumor microenvironment that are crucial for cancer initiation, progression and metastases formation [14, 15]. These regulatory mechanisms that operate in the tumor microenvironment serve to suppress the anti-tumorigenic effect of the Cytotoxic T (Tc) cells and the Type I T-helper (T_H1_) cells. This immune-suppressed tumor microenvironment acts as the ‘soil’ that nourishes and augments the growth of both the drug-sensitive as well as the drug-resistant sub-populations of the tumor, thereby posing a further challenge to the therapeutic strategies adopted to control cancer [16]. However, literature evidences showing the presence of a few tumor associated antigens (TAA) that helps in the recognition of these tumor cell sub-populations by the infiltrated T cells and the generation of effective immune responses upon Dendritic Cell (DC) vaccination throws light on the possibility of control of the disease using immunotherapy [17]. Hence a thorough understanding of the tumor-immune interaction, considering tumor sub-populations, is crucial to overcome the immune-suppression induced by the tumor.

In order to gain insight into the regulatory mechanisms governing these tumor-immune interactions, several studies have been performed using both theoretical as well as experimental techniques. Such studies have clearly indicated the role of Tc cells and IFN-γ in controlling Cancer progression [18]. Recent findings have suggested that synergistic activation of Tc cells and γδ-T cells are efficacious against HMLER-derived Breast Cancer stem-like cells, where γδ-T cells act as an early source of IFN-γ in tumor immunity, under special *in vitr*o conditions [19]. The development of Chimeric Antigen Receptor (CAR) T-cell has opened up new avenues for research in tumor immunity [20]. However, lack of truly CSC specific markers leads to on-target/off-tumor toxicity, where the CAR-T cells or any other CSC-targeted therapy kills the normal cells as well that display the same markers as that of CSCs [20, 21].

Mathematical models have been useful in delineating the multiplicity of the complex interactions governing the dynamics of the tumor-immune interaction that remains elusive through *in-vitro* experiments. In this context, *in-silico* studies have shown light on the CSC differentiation pattern and its effect on the tumor growth dynamics [3]. Here it has been observed that symmetric stem cell division shows a correlation with cancer progression [3]. This is in contrast to another report that mentions symmetric stem cell division lowers cancer risk as it reduces the accumulation of cellular damage [22]. However, the effect of CSC differentiation on drug-resistivity and the outcome of the interaction of these differentiated cells with the tumor microenvironment have not been explored sufficiently. On the other hand, models on tumor-immune interaction considering the involvement of tumor, immune effector cell and IL2 have enhanced our understanding about oscillations in tumor sizes, long-term tumor relapse and the conditions under which tumor elimination may be achieved using Adoptive Cellular Immunotherapy [23]. Mathematical models are now being exploited for the study of the efficacy of adaptive immunity for the elimination of aggressive tumors [24, 25], the existence of an angiogenic switch that regulates Cancer progression [26] and as a powerful tool in the design of optimal control strategies for Cancer [27, 28].

However, the study of the CSC differentiation pattern and the outcome of the interaction of these heterogeneous tumor cell sub-populations with the immune cells and cytokines present in the microenvironment is a challenge yet to be achieved in both experiments as well as modelling studies. With the aim to gain a clearer and unambiguous picture of the regulatory mechanisms involved in the immune-escape mechanism of the tumor cells, we propose an ODE-based mathematical model of the tumor-immune interaction that captures the development of a malignant tumor from the ‘seed’, the CSCs, and its interaction with the ‘soil’, the tumor microenvironment. In this model, we consider the three different modes of CSC differentiation, as well as the effect of random mutations and ask the question, how the stem cell differentiation patterns regulate the different cellular sub-populations in the tumor and how it affects the development of drug resistance? Using this model we have tried to address the unresolved question of the correlation of M2 macrophages with more resistant tumors by exploring the regulatory feedback loops that govern the dynamics of the tumor-sub-population and the roles of the cytokine feedbacks in shaping the tumor microenvironment. Prior to these studies, the model has been calibrated and the unknown parameters of the model have been estimated by fitting the initial growth kinetics of the model with data obtained from Gastric Cancer cell line using the MCMC-DRAM algorithm [29]. Moreover, the steady state behaviors of all the model variables have been quantifiably validated with previously reported experimental data obtained from cytometric and protein expression studies from both *in-vitro* studies as well as data obtained from different Cancer patients to establish the generic behavior of our model and ensure its acceptability in the design of treatment strategies.

In order to design treatment protocols for triggering the Cancer remission, we have introduced radio and chemotherapeutic strategies and observed the fold changes in the tumor mass in the presence and in the absence of resistant cells, where we demonstrate the failure of the conventional treatment strategies for curing Cancer. Thereafter, we have ventured the use of immunotherapy that has also been a popular choice for the elimination of the CSCs that are resistant to chemo and radio-therapeutic interventions [17, 30]. Hence, using the leads from our model analysis, we have attempted to propose combinatorial treatment strategies and design protocols that help in better suppression of the tumor, even in the presence of resistant cells. However, it may be mentioned here that a vital assumption in our model is that the drug-sensitive and drug-resistant population of tumor cells elicits similar immune responses. The resistant population of cells represents a fraction of the tumor cells that are unresponsive to the conventional treatment strategies. Thus, the difference in their behavior arises when the treatment/control is applied. Our novel modeling approach and strategy for the design of treatment protocol throws light on the ways to optimize drug schedules, dosage and treatment cycles required for the elimination of the tumor cells. This model may be used as a potential tool for the prediction of Cancer prognosis and calculation of fold changes in the tumor sub-populations in response to a new treatment regimen.

## 2. Model

**Fig 1:**
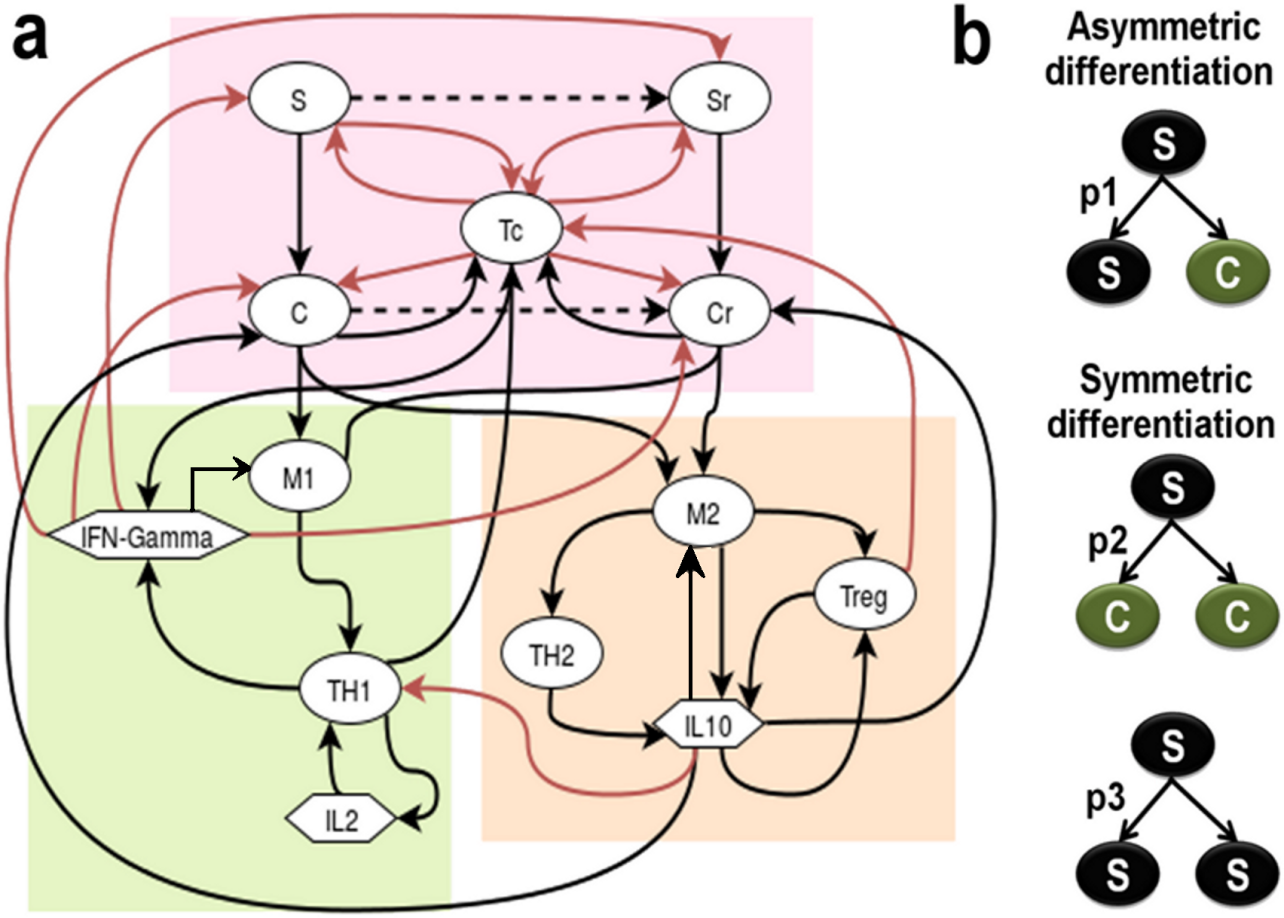
Diagrammatic representation of the tumor-immune interaction model. (a) Cellular interaction network representing the key players of the tumor microenvironment, viz. Cancer Stem Cells (S), Cancer cells (C), their drug resistant counterparts (S_R_ and C_R_), M1-TAM, M2-TAM, T_H1_, T_H2_, Tc, Treg immune cells, and cytokines IL10, IFN-γ and IL-2; The tumor microenvironment has been grouped into three parts, *viz*.- core tumor and infiltrated Tc cells (red box), the immune-stimulators (green box) and the immune-suppressors (orange box); the Black arrows represent Activation, while the Red arrow represent Inhibition; (b) Stem cell differentiation pattern.

The tumor-immune interaction model, depicted in Fig 1A, can be perceived as three regulatory modules – (i) the core tumor along with the tumor infiltrated Tc cells (red box), (ii) the immune-stimulators consisting of M1 cells, T_H1_ cells, IL2 and IFN-γ cytokines (green box) and (c) the immune-suppressors consisting of M2 cells, T_H2_ cell, Treg cells and IL10 cytokines (orange box). The interactions between these components of the model are based on known experimental evidences and immunological relevance. A detailed description of the model along with the mathematical assumptions, based on the biological phenomenon, used in its mathematical formulation has been described in the *Methods* section.

Based on these biological relevances and mathematical assumptions, the tumor-immune interaction network has been modelled using 13 Ordinary Differential Equations (Eqs 1–13) and 71 parameters (as enlisted in the *Methods* section). The state variables and parameters used in the formulation of the model have been listed in the S1 Text. The initial values of the model variables have been listed in the S1 Text. Here the initial value of the stem cell S_0_ = 1, while S_R_, C, C_R_ have been initialized as zero such that all the tumor sub-populations develop from the symmetric and asymmetric differentiation of a single stem cell that ensures the conservation of the stem cell hypothesis.

## 3. Therapeutic intervention

The protocols are designed using various combinations of *Radiotherapy*, *Chemotherapy* and *Immunotherapy*. The dosage, time duration and number of cycles for each therapy are varied to determine the optimal combination that gives us maximum fold changes in the tumor reduction as well as enhance the T_H1_/T_H2_ ratio to ensure better treatment efficacy. Two treatment protocols have been tested in our model. Protocol 1 is an adaptation from a previously reported protocol involving Chemotherapy and Radiotherapy, while Protocol 2 is novel combinatorial protocol proposed where we have introduced Immunotherapy by triggering the immune cells of our model (detailed discussion in the *Methods* section).

## 4. Results

### 4.1. Model validation with experimental data

#### 4.1.1. Tumor growth (without therapy)

**Fig 2:**
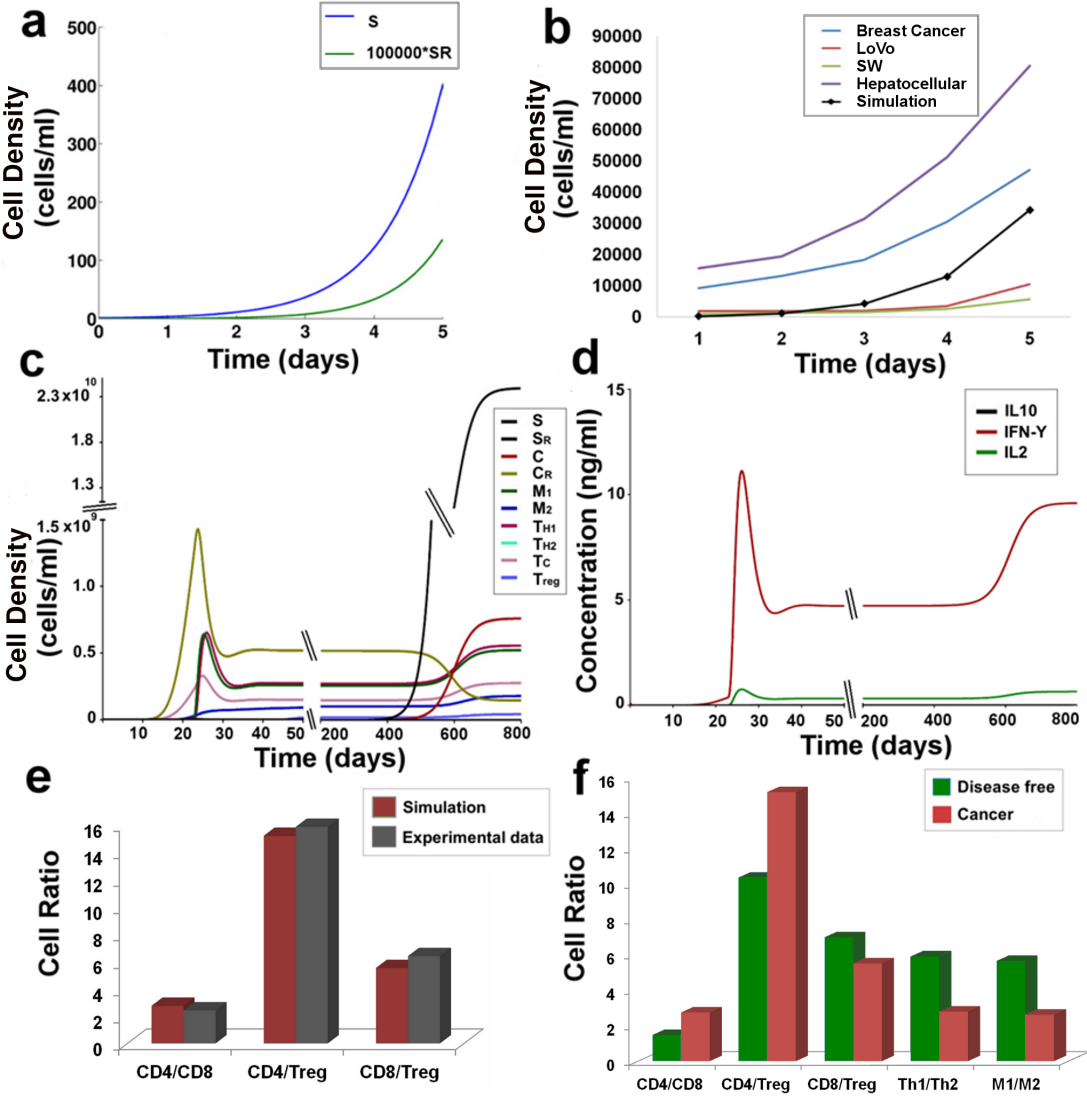
**Model validation with experimental data:** (a) Stem and Stem resistant cell proliferation at γ_S_ = 2 day^-1^ and δ_S_ = 0.2 day^-1^; (b) Proliferation of the Cancer Resistant cells as observed in experiments along with the observations from our simulation; figure depicts growth kinetics of the Breast cancer cell line MCF-7/TAX-resistant to Paclitaxel, Hepatocellular Carcinoma cell line SK-Hep1/CDDP3-resistant to Cisplatin, and Colon Cancer cell lines SW-620-L-OHP and LoVo-L-OHP-resistant to Oxaliplatin; (c) Temporal cellular behavior of the components of the model during tumor formation; (d) Temporal cytokine expression pattern during tumor formation; (e) Immune Cell Ratio at steady state- experiment versus simulation results (f) Immune cell ratio in the disease free condition versus cancer scenario. Note: CD4 = T_H1_ + T_H2_; CD8 = Tc.

The growth kinetics of the tumor is estimated by four variables of our model, S, S_R_, C and C_R_, signifying the four sub-populations of cells that are found in the tumor. In order to validate the growth kinetics of these tumor cell sub-populations of our model, we have used data from different experimental and theoretical studies of tumor growth estimation. The early temporal growth kinetics of the Stem (S) and Resistant Stem cells (S_R_) were validated over a period of 5 days, with the reports of Tomasetti and Levy [3], by choosing the parameter values γ_S_ = 2 day^-1^ and δ_S_ = 0.2 day^-1^ (Fig 2A). Here, it was observed that the Stem cells (S) start proliferating exponentially during this initial growth phase of tumor formation. During this time frame, the stem cells also start acquiring mutations and start producing the Resistant Stem Cells (S_R_) that gradually starts proliferating slowly and is maintained in very low numbers inside the tumor [3]. It is to be noted that for all our subsequent simulations we have used γ_S_ = 0.15 day^-1^ and δ_S_ = 2 x10^-7^day^-1^, as reported in the S1 Text.

Temporal behaviors of the Cancer (C) and Resistant Cancer (C_R_) sub-populations have been simulated to validate our model with experimental data (Fig 2B; See Methods *Section 7*.*7*). Fig 2B depicts the temporal growth kinetics of the Breast cancer cell line MCF-7/TAX-resistant to Paclitaxel [31], Hepatocellular Carcinoma cell line SK-Hep1/CDDP3-resistant to Cisplatin [32], and Colon Cancer cell lines SW-620-L-OHP and LoVo-L-OHP-resistant to Oxaliplatin [33]. Our simulation result mimics the average behavior of the resistant cancer cell lines over the time period of 5 days (Fig 2B), using the parameter set estimated through the MCMC method.

The model was simulated for a sufficiently long time to study the temporal evolution of the drug-sensitive and drug-resistant cancer cells without any therapeutic interventions (Fig 2C). Here, it was observed that during the early stages of tumor development, the stem cells (S) show a very slow rate of proliferation. According to our simulation results, it is observed that although a single stem cell initiates the formation of the entire tumor, the stem cells maintain a very low number during the first few months of tumor development. The rapid proliferation of the Cancer cells (C) during the early growth phase lead to their transformation to the resistant C_R_ species that soon start proliferating rapidly, thereby giving rise to a tumor. At the end of the exponential growth phase, the cancer progression is impeded by the activated M1, T_H1_ and Tc immune cells and the systems stay relatively stable for some time until the stem cells start proliferating exponentially and form the main bulk of the tumor. Our simulation results indicate that the first resistant stem cell of the tumor is detected at 400 days. Around 800 days the model reaches its steady state. The total tumor density at steady state can be estimated to be around 2.5x10^10^ cells/ml, i.e. 25 times higher than the reported minimum threshold of a clinically detectable tumor [3]. From here we calculate the relative abundance of the sub-populations of the tumor cells and derive that at steady state, the tumor is composed of 94.59% Stem cells (S), 4.49% Cancer cells (C), 1% Cancer Resistant cells (C_R_) and small fraction of Stem Resistant cells (S_R_) that comprises 0.001% of the tumor mass.

#### 4.1.2. Immune cell-ratio comparison with cytometric data

Our simulations results revealed the dynamics of the adaptive immune responses generated during the tumor development (Fig 2C). Here we observe that as the tumor sub-populations begins to proliferate, the Tc cells show enhanced activation that is required for the natural regression of the tumor (Fig 2C). However, as the tumor continues to proliferate and the resistant cancer cells (C_R_) peaks to 1.5x10^9^ cells/ml, there is a sharp rise in the M1 and T_H1_ cells proliferation. The combined effect of the Tc, M1 and T_H1_ cells helps to impede the tumor development and decrease it by 3 folds which then falls below the limit of tumor detection (i.e. 10^9^ cells/ml, [3]) and apparently stays dormant till 400 days. Thereafter, as the stem (S) and resistant stem (S_R_) cells start proliferating, the adaptive immunity becomes active again. However, this is also accompanied with the increase in abundance of M2 and Treg cells (Fig 2C), that helps in the sustenance and continued survival of the tumor cells.

In order to analyse the changes in the immune activation state before and after tumor formation, the immune cell ratio values obtained at the steady state are estimated and compared to the cell ratios in the normal disease-free condition (Fig 2F). It may be mentioned here, in Fig 2E and 2F, CD4 depicts the summation of both the T_H1_ and T_H2_ cells of our model, while CD8 implies Tc cells. From our model analysis, we observe that, during Cancer, the ratio of CD4 and CD8 cells reaches a mean value of 2.75, that is in sharp contrast to the normal healthy individuals which show a value of 1.48 (Fig 2F) [34]. On the other hand, the value of CD4:Treg ratio in Cancer shows a value of 15.2 that is higher than the ratio observed in the normal scenario. This happens due to the enhanced T_H2_ proliferation during tumor development. This also explains the reason for the elevated CD4/Treg ratio. However, the CD8:Treg ratio shows a decrease in Cancer patients and reaches to about 5.5, which is a characteristic of resistant tumors in mammals [35]. In Fig 2E we have compared these results of our numerical simulation with experimental data obtained from various literatures. Here, we clearly observe that our simulation corroborates very well with the experimental observations made from blood samples of Cancer patients (Fig 2E) [36, 37]. Additionally, we have observed the changes in the T_H1_:T_H2_ and M1:M2 ratios that have important implications in Cancer prognosis. We find that both T_H1_:T_H2_ ratio and M1:M2 ratio get decreased during Cancer as compared to the normal disease-free conditions (Fig 2F) [38, 39]. These results are in excellent agreement to the literature that suggests Cancer patients showing T_H1_:T_H2_ ratio below 8 show poor disease prognosis [39].

#### 4.1.3. Cytokine production

IL10 production is a characteristic feature for Cancer detection. During Cancer, the marked increase in IL10 production has been noted in blood samples of various cancer patients, where an average concentration of 0.01ng/ml has been recorded in various protein expression studies [40–42]. The temporal protein expression profile, from our simulations, suggests the IL10 expression starts increasing around the 15^th^ day until it reaches to a concentration of 0.005 ng/ml (Fig 2D). The IFN-γ production begins along with the proliferation of the Tc cells and increases sharply with the activation of the T_H1_ cells (Fig 2D). This is accompanied by IL2 production that helps in the continued proliferation of the T_H1_ cells. After the proliferation of the stem cells, the cytokine production increases further. The IL10 concentration starts increasing rapidly and attains a concentration of 0.009 ng/ml at steady state. The steady state concentrations of IFN-γ and IL2 reach 9.6 ng/ml and 0.6 ng/ml respectively. The cytokine expression levels from our simulation lie close to the experimentally observed ranges of protein expression of cancer treatment prior to their treatment [40].

### 4.2. Model analysis

#### 4.2.1. Development of drug resistance is governed by the pattern of stem cell differentiation

**Fig 3:**
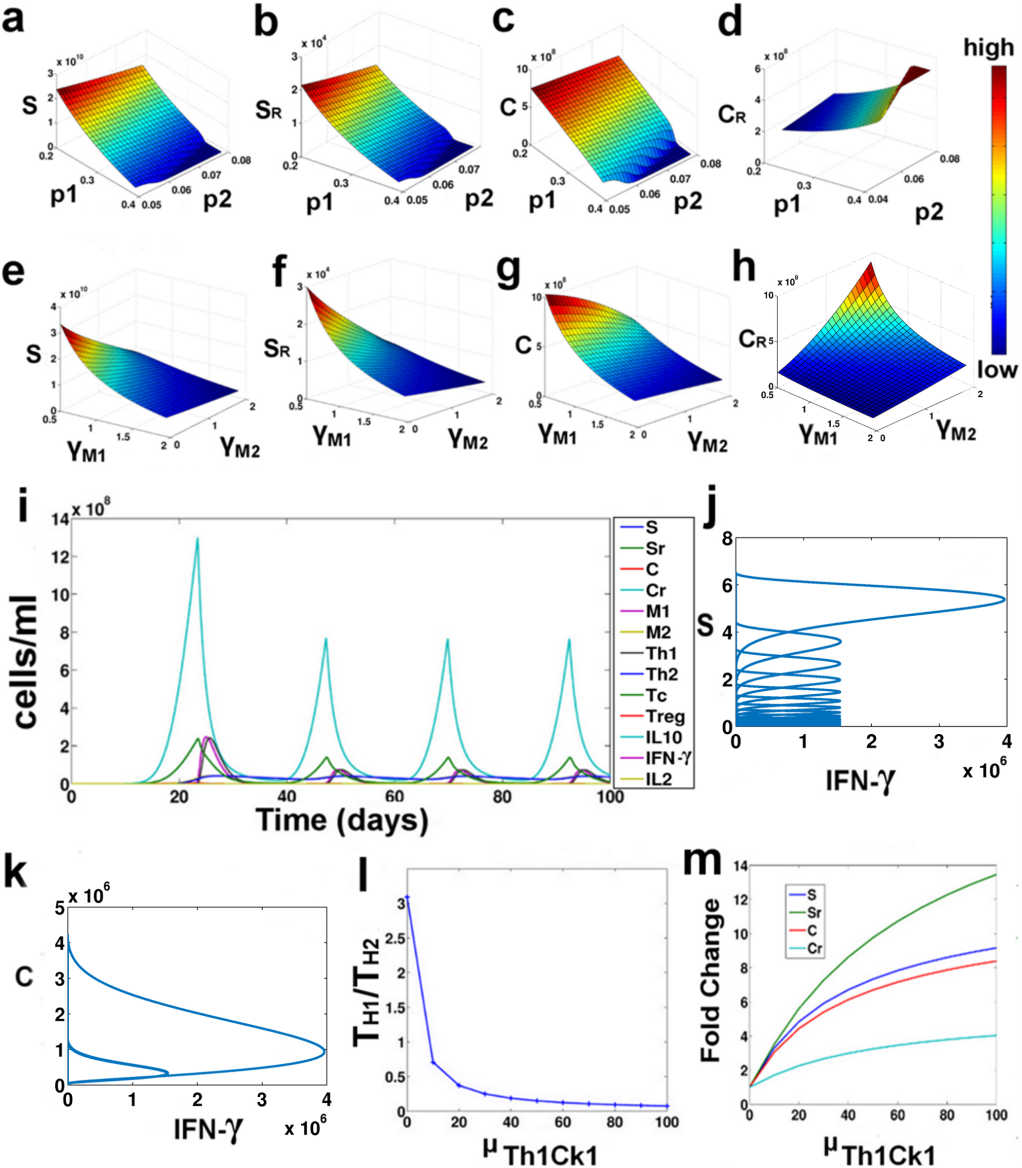
Parameter variation study. (a-d) Surface plot of the steady state values of S, S_R_, C and C_R_ under varying p1 and p2; (e-h) Surface plot of the steady state values of S, S_R_, C and C_R_ under varying γ_M1_ and γ_M2_; (i) Temporal Plot at β_Th1Ck2_ = 0.1; (j-k) Phase plane of S vs. IFN-γ and C vs. IFN-γ at β_Th1Ck2_ = 0.1; (l) T_H1_/T_H2_ ratio at varying μ_Th1Ck1_; (m) Fold change in steady states at varying μ_Th1Ck1_.

With the assumption that the stem cells predominantly tend to renew their pool of stem cells, i.e. with a probability p3, we have varied the values of p1 and p2 to observe the effect of the asymmetric and symmetric differentiation of the stem cell on the development of drug resistance (Fig 3A–3D). Here it may be observed that as we increase the values of p1 and p2, the rate of the stem cell renewal decreases gradually, thereby leading to decrease in the steady state values of S and S_R_ (Fig 3A and 3B). However, in the case of C cells (Fig 3C), we observe that as we increase the value of p1, the steady state values of C decreases, whereas the variation of p2 has little effect on the steady states of C (Fig 3C). The steady state level of C_R_ on the other hand, is greatly influenced by p1 and p2 (Fig 3D). With the increase in the value of p1 and p2, the steady states value of C_R_ increases, signifying as the mode of stem cell differentiation changes, the tumor cell sub-populations tend to transform into the resistant Cancer cells. Hence, from our results, we may infer that higher asymmetric stem cell division may be associated with a high rate of drug resistance.

#### 4.2.2. Dual role of tumor associated macrophages

The differential regulatory behavior of the type I and type II TAMs on the tumor cells was studied by varying the γ_M1_ and γ_M2_ parameters, governing the growth rate of the M1 and M2 macrophages (Fig 3E–3H). Here it was observed, as we increase the birth rate of M1, the steady state values of all the sub-populations of the tumor decreases. However, on varying γ_M2_, we observe that although the S, S_R_ and C sub-populations show a decrease in the steady state values, the C_R_ population increases (Fig 3E–3H). This result corroborates with the experimental observations that indicate that while M1 macrophages may a have an important role in suppression of the tumor growth, a higher abundance of M2 macrophages may lead to poor disease prognosis [43]. From our model analysis, we infer that a higher proliferation of M2 TAMs leads to an increased accumulation of resistant cancer cells in the tumor. This is primarily because of the feedback regulations that govern the dynamics of the tumor-immune interaction network.

#### 4.2.3. IFN-gamma and IL10 feedbacks regulate Cancer progression

The cytokines are the key regulators of the Tumor-Immune interaction network. The IFN-γ produced by the T_H1_ cells helps in maintaining the steady state dynamics of the entire system. Parameter variation studies reveal that as we increase IFN-γ production from the T_H1_ cells by changing the value of β_Th1Ck2_ between 10^-7^ and 10^-2^ ng/cell/day, the S and S_R_ cells show a dampening oscillation in their temporal behavior and these stem cell populations gradually decrease to a very low value. On the other hand, with the increasing β_Th1Ck2_ values, the temporal behavior of C and C_R_ cell population changes from dampening to stable oscillations at β_Th1Ck2_ = 0.1 (Fig 3I). The increased production of IFN-γ leads to the rapid killing of the S and S_R_ populations, whose oscillations dampen with time resulting in complete elimination of the stem cells from the system (Fig 3J). However, the high rate of C proliferation balances out the negative feedback effect of the high IFN-γ production, which keeps oscillating the system (Fig 3K). The phase-plot depicts the feedback regulation that operates between the Cancer (C) cells and IFN-γ that regulates the Cancer relapse. As the Cancer proliferation reaches 4x10^6^ cells, the IFN-γ production starts increasing which reduces the Cancer proliferation. When the Cancer cells fall below 1x10^6^ cells, the IFN-γ production also starts decreasing. However, at low levels IFN-γ, the Cancer cells start proliferating again (Fig 3K). This leads the system into stable steady state oscillations.

Another important feedback regulation that is crucial for the determination of tumor progression is the negative feedback effect of the IL10 cytokine on the T_H1_ proliferation. This is governed by the parameter μ_Th1Ck1_. As the value of μ_Th1Ck1_ is increased, the T_H1_/T_H2_ ratio decreased rapidly (Fig 3L). This results in the further proliferation of all the tumor sub-populations, i.e., S, S_R_, C and C_R_, and the fold changes in the steady state values of all four increases with increasing μ_Th1Ck1_ values (Fig 3M). Here it may be observed that inhibition of T_H1_ cells by IL10, results in higher fold changes of the steady state of S_R_ cells.

### 4.3. Development of treatment strategies

#### 4.3.1. Failure of chemo and radiotherapies due to the presence of resistant cells

**Fig 4:**
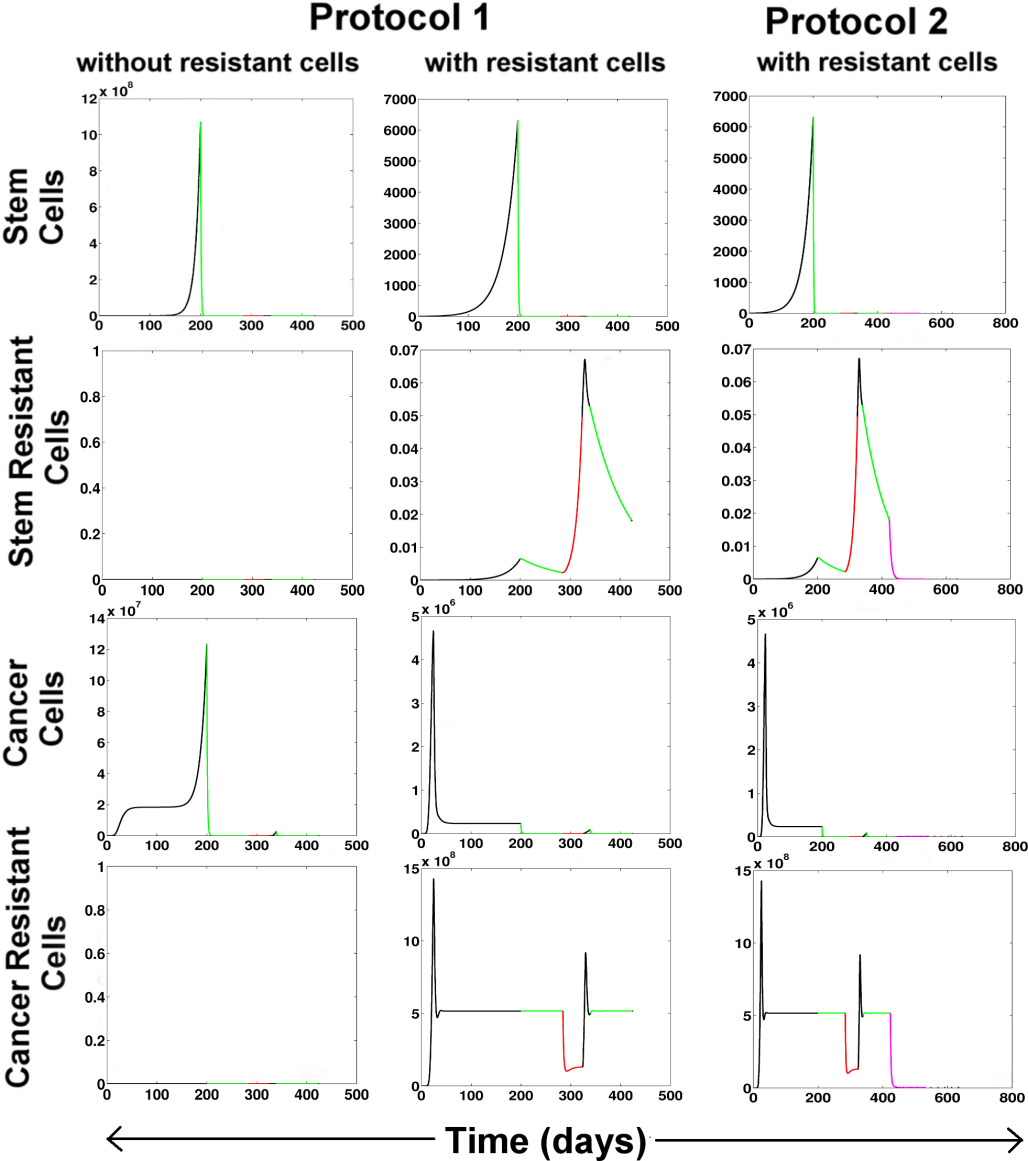
Changes in the tumor growth after therapeutic interventions. Protocol 1 has been applied without and with the presence of resistant cells. Protocol 2 efficiently suppresses tumor in spite of the presence of resistant cells. Color code: Black-without treatment, Green-Chemotherapy, Red-Radiotherapy, Pink-Immunotherapy.

Chemotherapy and Radiotherapy are effective for controlling tumor proliferation in the absence of the resistant cells, i.e. when the rate of transformation of the stem and cancer cells to their resistant counterparts reduces. Here we have tried to simulate the cancer scenario without any mutational pressure, i.e. m_C_ = 0 and m_S_ = 0. Under such conditions, when we apply the Treatment Protocol 1, we observe the Stem and the Cancer cells population decreases rapidly and an overall reduction in the tumor population is observed (Fig 4). However, during the formation of a tumor, a certain fraction of the tumor cells acquire resistance to drugs. Under such conditions, i.e. m_C_>0 and m_S_>0, when the Treatment Protocol 1 is applied at the end of the detection time (DT = 200 days), we observe that even though the drug-sensitive populations *viz*. S and C decreases, the resistant populations S_R_ and C_R_ remain unaffected during the chemotherapeutic cycles. Thereafter, during the Radiotherapy cycles, the S_R_ cell population being completely unaffected by radiation proliferates rapidly, while the C and C_R_ population sharply decreases for some time and then becomes stable. In the next treatment-free stage, S_R_, C and C_R_ start proliferating again. This activates the IFN-γ from the T_H1_ and Tc cells that help to bring down the S_R_ and C_R_ populations a little, that are then sustained at by the M2 and the Treg cells of the tumor microenvironment. The last phase of Chemotherapy does not have any effect on S_R_ and C_R_ populations. Hence the reduction in the overall tumor mass is not substantial. Also, it may be noticed here that at the end of this treatment regimen the T_H1_/T_H2_ ratio is reduced to 2.5 that is indicative of poor disease prognosis.

#### 4.3.2. Immune interventions for effective tumor remission

Combinatorial treatment protocol was designed to reduce the tumor burden and restore healthy T_H1_/T_H2_ ratio. Parameter variation studies revealed the importance of the T_C_ and the T_H1_ cells in the regulation of the steady state levels of the tumor cells. Here, Immunotherapy was introduced as two control variables, i.e., u3_Tc and u3_T_H1_, which boost the production of the T_C_ and T_H1_ respectively. The stimulus to the T_H1_ and T_C_ cells was started at the end of the last chemotherapeutic cycle and was administered for 20 days followed by 1 day rest. This was repeated for 10 cycles. The dosage of each therapy was varied in wide ranges, and it was observed that when Immunotherapy is low, the change in Radio and Chemotherapies does not affect the tumor population significantly which is reflected in the very small tumor fold change and low T_H1_/T_H2_ ratio (S1 Fig). As the Immunotherapy is increased, the fold change of tumor population increases along with the T_H1_/T_H2._ However at very high doses of immunostimulation, the fold changes decreases and the T_H1_/T_H2_ ratio increases abruptly that leads to extreme suppression of the T_H2_ cells in the system. Hence the region 1.5< d_I_ <2.5 can be considered as the ideal dosage of immunostimulation required for triggering the remission of Tumor. Using the leads from this analysis, the Protocol 2 was designed. At the end of this treatment regimen, it was observed that all the four tumor sub-populations showed a huge reduction in their proliferation, i.e. 136 fold reduction in tumor mass (Protocol 2). The T_H1_/T_H2_ ratio was boosted to 8.8.

## 5. Discussion

The model developed here throws light into the development of a full grown tumor from a single cancer stem cell (S), and the influence of the tumor microenvironment during its maturation. The study of the temporal evolution of tumor development shows that although the cancer stem cell forms the ‘seed’ from which the tumor emanate, the stem cell population remains low in the beginning. These cancer stem cells, owing to their slow replication, are intrinsically resistant to radiotherapy and are only partially sensitive to chemotherapy [44, 45]. Additionally, the stem cell sub-populations have a strong immune-suppressive effect on the tumor microenvironment [46]. This phenomenon has been captured in our model in the study of the temporal evolution of the tumor-immune interaction dynamics, where we observe that coincident with the proliferation of the stem and resistant stem cell there is also an increased proliferation of the M2 and Treg cells (Fig 2C). This consequently leads to the lowering of the M1/M2 and T_H1_/T_H2_ ratio that is associated with the formation of resistant tumors (Fig 2F). Moreover, it has been observed in our study that the proliferation of stem cell sub-populations leads to suppression of the Tc cells and the activation of the Treg cells, that results in the lowering the CD8/Treg ratio during Cancer (Fig 2F). This happens primarily because of the direct negative regulatory effects of the stem and resistant stem cells on the growth of the Tc cells. Hence, an early detection of the tumor is crucial for an effective treatment, when the stem cell population in the tumor remains low and the resistant stem cell population is not yet formed.

The model also captures the different patterns of CSC differentiation and its role in determining the fate of the tumor. Here, it has been observed that as the differentiation pattern of CSC shifts towards the asymmetric pattern, the CSC pool begins to deplete and the CSC starts producing the terminally differentiated cancer cells that have a finite lifespan. The reduction in the stem cell population helps in the reduction of its immune-suppressive effects on the Tc cells. At the same time, the differentiation of the stem into the cancer cells stimulates the Tc cells to get activated that now inhibit the tumor via the negative feedback regulation. However, the steady state value of the resistant cancer cells increases and overrides the negative feedback effect of the immune cells, reinforcing the observations that a higher asymmetric stem cell differentiation may be associated with the formation of more resistant tumors. Our model analysis also indicates that at low p_1_ value, as the probability of symmetric differentiation (p_2_) of stem cells is increased, the steady state levels of stem cells rapidly decreases, however it has little effect on the steady state value of Cancer cells. On the contrary, at high p_1_ value, the increase in p_2_ leads to the transformation of the cancer to resistant cancer cells. These results signify that reduction in stem cell symmetric renewal (p3) of the cell leads to its differentiation into more resistant tumors.

**Fig 5:**
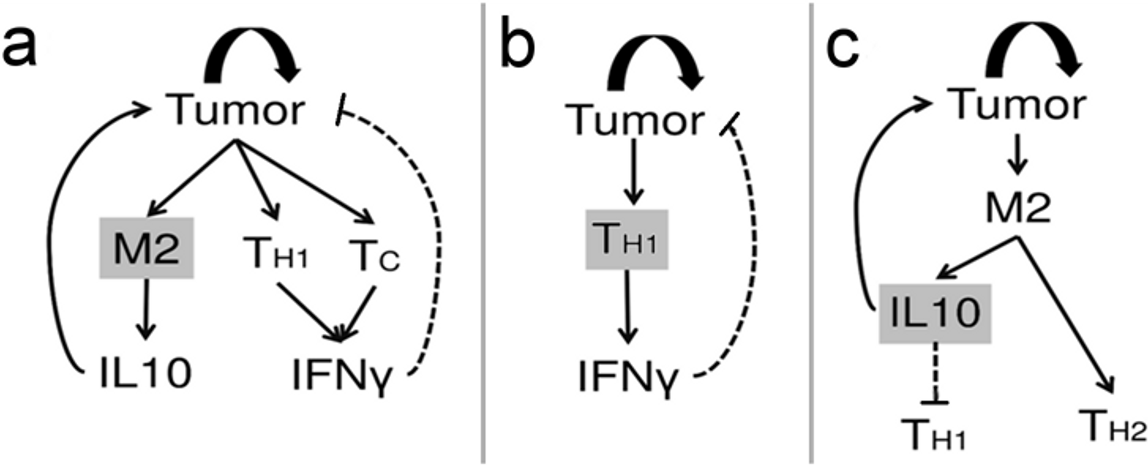
Regulatory feedback loops. (a) The M2 macrophage triggers two feedback loops. The first loop through IL10 is a positive feedback that triggers tumor proliferation. This, in turn, activates the second loop through IFN- γ that inhibits the tumor; (b) The T_H1_ derived IFN- γ inhibits the tumor via a negative feedback that leads to an oscillation in the population of tumor cells; (c) The positive feedback loop through IL10 is responsible for the maintenance of tumor and suppression of T_H1_/ T_H2_ ratio.

The model further elucidates a dual role of the M2-TAMs in regulating the tumor formation (Fig 3E–3H). Here we observe that on one hand, the M2-TAMs aid in the suppression of the S, S_R_ and C cells of the tumor. This is because M2 is a prime source for the production of IL10 cytokine that has an important role in positively regulating the proliferation of the Cancer (C) cells. Hence, as the M2-TAMs increase in abundance, the cancer cells begin to proliferate via a positive feedback loop (Fig 5A). This leads to the activation of both the Tc and T_H1_ cells that inhibits the tumor cells *via* their negative feedback by producing a higher amount of IFN-γ and higher cytotoxic activity of the Tc cells (Fig 5A). However, on the other hand, it may be observed that M2-TAMs help in the growth of the C_R_ cells via the positive feedback loop, while the negative feedback has little effect on the C_R_ sub-population. This observation explains the reason for the refractory behavior of the tumor to treatment strategies under the presence of the M2-TAMs [47].

Our study reveals the functional behavior of the feedback mechanisms that regulate the behavior of the entire tumor-immune interaction network. From our parameter variation studies, the importance of the IFN-γ production in regulating the temporal behavior of the tumor development is observed. Here, we found that as the rate of IFN-γ production from the T_H1_ cells increases, the negative feedback effect of IFN-γ on the tumor helps in the suppression of the tumor cells (Figs 3I and 5B). However, the replicative potential of the Cancer and resistant Cancer balances out the negative effect of the IFN-γ leading to an oscillatory tumor-relapsing behavior, whereas the slowly replicating stem and resistant cells sub-populations gradually get eliminated from the tumor due to the high production of the IFN-γ cytokine (Fig 3J and 3K). This finding has important implications in the design of Immunotherapeutic protocols, where we observe the differential behavior of the tumor cells in response to high IFN-γ production. Using our model, we further explore the effects of the negative feedback of the IL10 cytokine on the T_H1_ cells (Figs 3L, 3M and 5C). We also make an important observation, where we find that as the sensitivity of T_H1_ cells to IL10 increases, the T_H1_/T_H2_ ratio decreases sharply. This leads to the increased proliferation of the tumor cells. The fold change in the steady values of the resistant stem cells is the greatest signifying the prognostic role of the T_H1_/T_H2_ ratio in predicting tumor progression and the formation of a resistant tumor with higher proportion of resistant stem cells.

With the knowledge of the regulatory mechanisms governing the differential response of the tumor sub-populations to the microenvironment, we have tried to explore the effect of treatment strategies conventionally adopted for the treatment of Cancer (Fig 4). Using our model we have been able to show that the reason for the failure of conventional Chemotherapy and Radiotherapy is primarily due to the formation of the resistant cancer stem cells S_R_ and resistant cancer cells C_R_ within the tumor. Under the conditions where there are no mutations conferring drug resistivity to the tumor, a significant reduction in tumor mass may be achieved using Chemo and Radio therapy alone. However, in reality, a small population of resistant tumor cells exists in the tumor that remains refractory to these treatment protocols. In order to successfully remove the tumor, a combination of the conventional treatment protocol along with Immuno-therapy can help alleviate the disease scenario. There can be several ways of boosting the immune system. However, in this work, we propose that a synergistic stimulus to both T_H1_ and Tc cells is required for the generation of an adaptive immune response that is capable of reducing both the drug-sensitive as well as the drug-resistant sub-populations of the tumor. In order to achieve this, dosage of Radio, Chemo and Immunotherapies were varied to create 1000 different treatment combinations and thereafter a treatment protocol (Protocol 2) has been designed to ensure maximal reduction in the tumor mass, as well as the restoration of a healthy T_H1_/T_H2_ balance.

In this model, we try to capture the high complexity of the tumor microenvironment with a simple ODE model that represents the interaction of the tumor cell sub-populations and the immune cells at the phenotypic level. Here we assume that the parameters governing these cellular interactions are a cumulative outcome of the various molecular and the intracellular signalling events occurring in the microenvironment that influence the immune evasion but have not been explicitly considered in this model for its simplification. However, it is worth mentioning that depending on the availability of data and hybrid modeling techniques involving a combination of different mathematical tools and strategies, this model may further be improvised by considering the effect of the various molecular events such as angiogenesis, the role of the miRNA, exosomes and chemokines in mediating the cellular interactions, the metabolic pathways as well as the hypoxic conditions [48], that may further help us unravel unknown regulations underlying the tumor immune interaction and the development of drug-resistance.

## 6. Conclusion

With respect to the long standing ‘seed and soil’ hypothesis, we propose a model that throws light into the previously unexplored regulations governing tumor-immune interaction. This novel approach of developing of a tumor-immune interaction model considering both the stem cell differentiation pattern as well as the effect of the microenvironment has helped us in unveiling the effect of stem cell differentiation on the development of drug resistance and the different mechanistic regulations governing the tumor-immune interaction dynamics. However, this model does not capture the diffusion kinetics of the cytokines or the time delay associated with the cytokine regulations. Nonetheless, the observations derived from the model have been corroborated extensively with the experimental observations in cytometric and protein expression studies that strengthen the reliability of our model for the prediction of mechanistic regulations of tumor-immune interaction and design of the treatment protocols. This study can further be used to optimize treatment strategies, drug dosage and time schedules for designing advanced treatment protocols for Cancer.

## 7. Methods

### 7.1. Model development

#### 7.1.1. Tumor formation

The core tumor consists of the Cancer Stem Cells (S), the Cancer Cells (C) and their drug resistant counterparts Resistant Stem Cells (S_R_) and Resistant Cancer Cells (C_R_) (Fig 1A, red box). The model takes into consideration the different patterns of stem cell differentiation, *viz*. the symmetric and asymmetric stem cell differentiation (Fig 1B). In the asymmetric differentiation, one stem cell (S) produces one daughter stem cell (S) and a differentiated progenitor Cancer cell (C) with probability p_1_, while in the symmetric differentiation one stem cell (S) produce either two Cancer cells (C) with probability p_2_ or two stem cells (S) with probability p_3_ (where, p_1_+p_2_+p_3_ = 1). The stem cells undergoing asymmetric differentiation acquire mutation (represented with black dotted line in Fig 1A) with a probability m_S_ that leads to the transformation of a stem cell (S) to a resistant stem cell (S_R_). Since the probability that this mutation hits the daughter stem cell and not the differentiated cancer cell is 0.5, the probability of formation of S_R_ from S is further multiplied by p_1_/2. The symmetric differentiation leads to the renewal of the non-mutant stem cell (S) pool with a probability (1-m_S_)(1-p_1_-p_2_) [3]. Considering these factors (as described by Tomasetti and Levy [3]), we assume that the growth rate of S can be mathematically represented as (γ_S_(1 − m_S_)(1 − p_1_ − p_2_))S, while the rate of depletion of the stem cell pool, that includes the differentiation of S to C and the transformation S to S_R_ can be represented as $\left(\delta_{S}+\left(p_{2}*\gamma_{S}\right)+\gamma_{S}*\frac{m_{S}*p_{1}}{2}\right)S$. It may be mentioned here that γ_S_ and δ_S_ represents that natural birth and death rates of S cells. A similar nomenclature has been followed for all the other cell types.

The resistant stem cells are formed from the transformation of an S to S_R_. The S_R_ follows a similar pattern of self-renewal and differentiation that leads to the replenishment of the S_R_ pool and the formation of differentiated C_R_ cells [3]. Here, it may be assumed that the S_R_ represents the compartment of stem cells that accumulate all the mutations in its pool, such that no separate compartment for any secondary mutations has been considered here in this model.

The Cancer cells (C) are formed from the stem cells (S) with the probability p_1_+p_2_. These C cells follow a Gompertzian growth kinetics that can be mathematically represented by $\gamma_{C}*\log\;\left(\frac{C_{max}}{C+r_{1}}\right)$, where C_max_ is the carrying capacity of the tumor [49]. Here, it has been assumed that during proliferation these C cells acquire mutations with a probability m_C_ and get transformed into C_R_ cells. Hence the probability of proliferation of the non-mutant C cell is further multiplied by a factor (1-m_C_). The C_R_ cells are formed from the differentiation of the S_R_ cells with probability p_1_+p_2_ and the transformation of the C to C_R_ cells with probability m_C_. The C_R_ cells also follow similar Gompertzian growth kinetics. The total carrying capacity of these non-stem tumor cells has been considered as K_tumor_, while each of C and C_R_ has a carrying capacity of K_tumor_/2, so that both the cell populations can use the nutrients equally and have an equal advantage in proliferation.

#### 7.1.2. Immune cells in the tumor microenvironment

As the tumor develops the resident TAMs, both M1 and M2, encounters the C and C_R_ cells of the tumor and gets activated (Fig 1A) [38]. It may be assumed that this cell to cell interaction will follow a saturating kinetics where even in the presence of a high number of tumor cells, the availability of TAMs acts as the limiting condition. Hence a Michaelis-Menten type functional form may be used to represent the TAM activation, e.g. $\gamma_{M1}*\left(\frac{M_{1}*(C+C_{R})}{M_{1}+\lambda_{M1}}\right)$. These M1 and M2 TAMs now activate the T_H1_ and T_H2_ cells respectively [50]. Here the abundance of the T_H_ cells acts as the limiting condition. In a similar way, the M2-TAMs also activate the Treg cells present in the tumor microenvironment [51]. The Tc cells, on the other hand, infiltrate the tumor, gets directly activated by C and C_R_ cells (Fig 1A). However, the S and S_R_ cells of the tumor inhibit the Tc cell proliferation [9]. This is a bidirectional reaction, as the activated Tc also tries to kill the tumor cell sub-populations via its cytotoxic activity [52, 53]. The Treg cells act as immune-suppressor of the system and try to inhibit Tc proliferation, whereas the T_H1_ cells act as immune-stimulator of the system that helps in Tc proliferation and tumor infiltration [54, 55]. All these cell to cell interactions tend to follow saturation growth kinetics and hence have been modelled using the Michaelis Menten form discussed earlier [23].

#### 7.1.3. Cytokines and feedbacks

Tumor formation triggers the immune system to produce cytokines. In this model, three important cytokines have been considered, *viz*. IFN-γ, IL-2 and IL-10 (Fig 1A). The activation of the T_H1_ cells stimulates the production of IL-2 cytokine from them. The amount of cytokine produced is directly proportional to the abundance of effector cells activated. Hence, this has been modelled using the Law of Mass Action, e.g. (*β*_*Th*1*CK*3_ * *T*_*H*1)_, where β_Th1CK3_ (units: ng cell^-1^day^-1^) is the rate of production of IL2 from T_H1_ cells [56]. This IL2 is responsible for the auto-regulation and sustained proliferation of the T_H1_ cells. Hence, we have considered a positive feedback loop from IL-2 to T_H1_ cell that has been modelled using a saturating function $\frac{\mu_{Th1Ck3}*IL2*T_{H1}}{IL2+k9}$, where the cytokine acts as the limiting factor [23, 56]. Similarly, the IFN-γ is produced by T_H1_ and Tc cells which have a negative feedback effect on all the tumor cell sub-populations. The production of IL-10 is regulated by M2, Treg and T_H2_ cells. An auto-regulatory positive feedback loop exists between IL10 and the Treg cells. IL10 also plays an important role in the proliferation of the C and C_R_ cells and inhibition of the T_H1_ cells. This IL10 mediated regulation captures the inhibitory actions of T_H2_ on T_H1_ cells that are often observed in Cancer scenario.

### 7.2. Model equations

Based on the biological relevance and mathematical assumptions discussed above, the equations representing the tumor-immune interaction network comprising of 13 Ordinary Differential Equations (Eqs 1–13) and 71 parameters have been enlisted below:

$$•\;\frac{\mathrm{dS}}{\mathrm{dt}}=\left(\gamma_{S}\left(1-m_{S}\right)\left(1-p_{1}-p_{2}\right)\right)S-\left(\delta_{S}+\left(p_{2}{*\gamma }_{S}\right)+\gamma_{S}*\frac{m_{S}{*p}_{1}}{2}\right)\;S-\left(\frac{\mu_{S}*S*\mathrm{IFN}\gamma }{k_{1}+\mathrm{IFN}\gamma }\right)–\left(\frac{\mathrm{tck}*S*\mathrm{Tc}}{\mathrm{ktc}1+\mathrm{Tc}}\right)$$ (Eq 1)

$$•\;\frac{{\mathrm{dS}}_{R}}{\mathrm{dt}}=\left(\gamma_{S}\left(1-p_{1}-p_{2}\right)-\left(\delta_{S}+(p_{2}{*\gamma }_{S})\right)\right)\;S_{R}+m_{S}{*\gamma }_{S}*\left(1-\frac{p_{1}}{2}-p_{2}\right)\;S-\left(\frac{\mu_{\mathrm{SR}}{*S}_{R}*\mathrm{IFN}\gamma }{k_{2}+\mathrm{IFN}\gamma }\right)–\left(\frac{\mathrm{tck}*\mathrm{Sr}*\mathrm{Tc}}{\mathrm{ktc}2+\mathrm{Tc}}\right)$$ (Eq 2)

$$•\;\frac{\mathrm{dC}}{\mathrm{dt}}=\gamma_{C}*\left(1-m_{C}\right)*\log\;\left(\frac{0.5*K_{tumor}}{C+r_{1}}\right)*C+\gamma_{S}*\left(p_{1}+p_{2}\right)*S–\delta_{C}*C-m_{C}{*\gamma }_{C}*C+\left(\frac{\mu_{C1}*C*\mathrm{IL}10}{\mathrm{IL}10+k_{3}}\right)-\left(\frac{\mu_{C2}*C*\mathrm{IFN}\gamma }{\mathrm{IFN}\gamma +k_{4}}\right)–\left(\frac{\mathrm{tck}*C*\mathrm{Tc}}{\mathrm{ktc}3+\mathrm{Tc}}\right)$$ (Eq 3)

$$•\;\frac{dC_{R}}{dt}=\gamma_{C}*C_{R}*log\;\left(\frac{0.5*K_{tumor}}{C_{R}+r_{2}}\right)+\gamma_{S}*S_{R}*\left(p_{1}+p_{2}\right)+m_{C}*\gamma_{C}*C–\delta_{CR}*C_{R}+\left(\frac{\mu_{C1}*C_{R}*IL10}{IL10+k_{5}}\right)-\left(\frac{\mu_{C2}*C_{R}*IFN\gamma }{IFNgamma+k_{6}}\right)–\left(\frac{tck*Cr*Tc}{ktc4+Tc}\right)$$ (Eq 4)

$$•\;\frac{dM_{1}}{dt}=\gamma_{M1}*M_{1}*\left(\frac{C+C_{R}}{M_{1}+\lambda_{M1}}\right)–\delta_{M1}*M_{1}+\left(\frac{\mu_{M1Ck2}*M1*IFN\gamma }{IFN\gamma +k_{7}}\right)$$ (Eq 5)

$$•\;\frac{dM_{2}}{dt}=\gamma_{M2}*M_{2}*\left(\frac{C+C_{R}}{M_{2}+\lambda_{M2}}\right)-\delta_{M2}*M_{2}+\left(\frac{\mu_{M2Ck1}*M2*IL10}{IL10+k_{10}}\right)$$ (Eq 6)

$$•\;\frac{dT_{H1}}{dt}=\gamma_{TH1}*\left(\frac{T_{H1}*M_{1}}{\lambda_{TH1}+T_{H1}}\right)–\left(\delta_{TH1}*T_{H1}\right)–\frac{\mu_{TH1Ck1}*IL10*T_{H1}}{IL10+k_{8}}+\frac{\mu_{Th1Ck3}*IL2*T_{H1}}{IL2+k9}$$ (Eq 7)

$$•\;\frac{dT_{H2}}{dt}=\gamma_{TH2}*\left(\frac{T_{H2}*M_{2}}{\lambda_{TH2}+T_{H2}}\right)–\left(\delta_{TH2}*T_{H2}\right)$$ (Eq 8)

$$•\;\frac{dTc}{dt}=\gamma_{Tc}*Tc*\left(\frac{C+C_{R}}{Tc+\lambda_{Tc1}}\right)+\gamma_{Tc}*\frac{Tc*T_{H1}}{Tc+\lambda_{Tc4}}-\mu_{TcS}*Tc*\left(\frac{S+S_{R}}{Tc+\lambda_{Tc2}}\right)–\delta_{Tc}*Tc-\mu_{TcTreg}*Tc*\left(\frac{T_{reg}}{\lambda_{Tc3}+T_{reg}}\right)$$ (Eq 9)

$$•\;\frac{dT_{reg}}{dt}=\gamma_{Treg}*\left(\frac{T_{reg}*M_{2}}{T_{reg}+\lambda_{Treg2}}\right)–\delta_{Treg}*T_{reg}+\left(\mu_{TregCk1}*\frac{IL10*T_{reg}}{T_{reg}+k_{11}}\right)$$ (Eq 10)

$$•\;\frac{dIL10}{dt}=\beta_{M2}*M_{2}–\delta_{Ck1}*IL10+\beta_{Treg}*T_{reg}+\beta_{Th2}*T_{H2}$$ (Eq 11)

$$•\;\frac{dIFN\gamma }{dt}=\beta_{Th1CK2}*T_{H1}+\beta_{Tc}*Tc–\delta_{Ck2}*IFN\gamma$$ (Eq 12)

$$•\;\frac{dIL2}{dt}=\beta_{Th1CK3}*T_{H1}-\delta_{Ck3}*IL2$$ (Eq 13)

### 7.3. Control and therapeutic strategies

- **Radiotherapy (R) -** With the aim to reduce the tumor cell proliferation, control variables were introduced in our model. Here, the control variable u1 signifies the probability of cell death due to Radiotherapy (Eq 14),
  $$u1=1‑\exp\left(‑{\alpha d}_{R}-{\beta d}_{R}{}^{2}\right)$$ (Eq 14)
  where α and β are the parameters governing the radio-sensitivity of the cells, and d_R_ is the dose of radiotherapy applied, measured in Grey (Gy) units [57]. The value of α and β depends on the oxygenation state of the cell [58]. In our model it has been considered that Radiotherapy affects only the Cancer (C) and the Cancer Resistant (Cr) populations of the tumor. It has no effect on the stem cells owing to their slow growth rate.
- **Chemotherapy (C)** - The control variable u2, signifying chemotherapy has an effect on the drug-sensitive stem (S) and cancer (C) cells of the tumor (Eq 15). u2_S and u2_C are defined as the probabilities of cell death, due to chemotherapy, of Stem cells and Cancer cells respectively (Eq 15 and Eq 16).
  $$u2\_S=f_{C}*\left(1‑\exp\left(‑M_{C}*d_{C}\right)\right)‑k_{S}$$ (Eq 15)
  $$u2\_C=f_{C}*\left(1‑\exp\left(‑M_{C}*d_{C}\right)\right)$$ (Eq 16)
  Here, f_C_ denotes the frequency of chemotherapy per day, M is defined as the efficiency of the chemotherapeutic drug in m^2^mg^-1^ denoting the area of the tumor affected per mg of the drug and d_C_ is the concentration of the drug in mg m^-2^. The efficacy of the chemotherapy of the stem cells depends on the factor k_S_ that represent the inhibitory effect of IL-4 on the stem cells that reduces the efficacy of the drugs. Sequestration of IL-4 makes the stem cells sensitive to chemotherapy [44, 59].
- **Immunotherapy (I) -** The parameter set was systematically screened to identify key parameters governing the negative feedback of the immune cells on the Tumor population. Then, the immunotherapy was introduced in our model as perturbations to the system in order to overcome the immunosuppressive effect of the tumor cells and to restore a healthy T_H1_ /T_H2_ balance.
  $$u3\_\mathrm{Tc}=d_{I}*M_{\mathrm{TC}}$$ (Eq 17)
  $$u3\_\mathrm{TH}1=d_{I}*M_{\mathrm{TH}1}$$ (Eq 18)
  Eq 17 and Eq 18 depicts the control variables for providing immune-boost to the T_C_ and T_H1_ cells, respectively. d_I_ signifies the dose of immunostimulant, measured in mg day^-1^ that must be given to the system, while M_TC_ and M_TH1_ are the measures of the sensitivity of the T_C_ and T_H1_ cells, respectively.

### 7.4. Designing treatment protocols

The protocols are designed using various combination of the above mentioned treatment strategies, viz. *Radiotherapy*, *Chemotherapy* and *Immunotherapy*. The dosage, time duration and number of cycles for each therapy are varied to determine the optimal combination that gives us maximum fold changes in the tumor reduction. The general form of the protocols can be described as follows:

$${DT}_{200}\to {{(R}_{tR}^{dR/n})}_{nR}\to {FT}_{tFT}\to {\left({Ch}_{tC}^{dC}\right)}_{nC}\to {\left(I_{tI}^{dI}\right)}_{nI}$$

Here, *Ch* denotes Chemotherapy, *R* denotes Radiotherapy and *FT* signifies a treatment-free period or relaxation time. The model was run till 200 days before the start of any therapeutic interventions. This has been considered as the standard detection time (*DT*) for a full grown tumor. Here the subscripts (t_R_, t_C_ and t_I_) denotes the time duration for which the treatment was given, and the superscripts (d_R_, d_C_ and d_I_) represent the dosage. The subscript outside the bracket (n_R_, n_C_ and t_I_) denotes the number of cycles for which that treatment was repeated.

#### 7.4.1. Protocol 1

This Protocol is an adaptation of the standard treatment protocol used for applying chemo and radiotherapy (adapted from British Columbia Cancer Agency Protocol GIGAJCPRT - http://www.bccancer.bc.ca/). It was applied to our model to observe the fold changes in the tumor cell population. The protocol can be summarized as follows:

$${DT}_{200}\to {\left({Ch}_{14}^{800}\right)}_{6}\to {(R}_{40}^{60/28})\to {FT}_{15}\to {\left({Ch}_{14}^{800}\right)}_{6}$$

#### 7.4.2. Protocol 2

This Protocol was designed as a combinatorial treatment protocol of Chemotherapy, Radiotherapy and Immunotherapy to enhance the treatment efficacy. Based on Protocol 1, this Protocol is an improvisation where Immunotherapy has been included that boosts both the T_H1_ and Tc simultaneously. In order to design this combinatorial treatment protocol, the dose of Radio, Chemo and Immunotherapy were varied over wide ranges in order to create 1000 treatment combinations. The treatment efficacy of each combination was plotted in a 4-dimensional scatter plot, measured in terms of fold change and T_H1_/T_H2_ ratio (S1 Fig). The Protocol 2 can be summarized as follows:

$${DT}_{200}\to {\left({Ch}_{14}^{800}\right)}_{6}\to {(R}_{40}^{60/28})\to {FT}_{15}\to {\left({Ch}_{14}^{800}\right)}_{6}\to {\left(I_{20}^{2}\right)}_{10}$$

#### 7.4.3. Measuring treatment efficacy

The efficacy of a treatment protocol is measured by the reduction in the size of the tumor and the overall recovery from the immune-suppression induced by the tumor, to ensure minimal chances of tumor relapse. Hence we have defined two parameters that can be used as an indicator of Cancer prognosis:

- **Fold Change** – The treatment efficacy was estimated by measuring the fold change of the tumor mass at the end of the treatment period as compared to the tumor mass measured at the time of detection.
- **T**_**H1**_**/T**_**H2**_ **ratio** - In order to ensure maximum treatment efficacy and minimize chances of Cancer relapse, the T_H1_/T_H2_ ratio was used as an indicator for disease prognosis. A minimum threshold of T_H1_/T_H2_ ≥ 5 was chosen to optimize treatment protocol.

### 7.5. Positivity and boundedness

This system of equations (Section 1.2, Eqs 1–13) can be analyzed with the initial conditions (S1 Text) defined in the thirteen dimensional variable space

$$R_{+}^{13}=[(S,S_{R},C,C_{R},M1,M2,T_{H1},T_{H2},Tc,Treg,IL10,IFN\gamma ,IL2)\in R^{13}|(S,S_{R},C,C_{R},M1,M2,T_{H1},T_{H2},Tc,Treg,IL10,IFN\gamma ,IL2)\geq 0]$$

It can be proven, that all solutions of the system in $R_{0+}^{13}$ remain in $R_{0+}^{13}$. Hence, $R_{0+}^{13}$ is positively invariant, and it is sufficient to consider solutions only in $R_{0+}^{13}$. In this region, the usual existence, uniqueness and continuation results hold for the system. From our numerical simulations also, we have observed the existence of positive solutions. The solution set we get for the set of ODEs represents the effective cell population and protein concentrations of the species considered in the model at different time points during the tumor development. The fixed point attained by all the variables of the model is a part of this positive solution space.

Also, we observe that the right-hand side of Eqs 1–13 (Section 1.2) are smooth functions of the variables (S, S_R_, C, C_R_, M1, M2, T_H1_, T_H2_, Tc, Treg, IL10, IFNγ, IL2). Also, since all the parameters are non-negative, local existence and uniqueness properties hold in $R_{+}^{13}$, and if the following necessary conditions are satisfied,

1. S_0_ > 0
2. p_1_+p_2_ < 1
3. m_S_ < 1
4. m_C_ < 1
5. γ_S_(1-p_2_) > δ_S_

then, we can state the following proposition.

**Proposition 1:** All the solutions of Eqs 1–13 (Section 1.2) which initiate in $R_{+}^{13}$ are uniformly bounded.

**Proof:** The proof of Proposition 1 is obvious as all the variables satisfy the condition of positive invariance for all the solutions of Eqs 1–13 (Section 1.2) which initiate in $R_{+}^{13}$, the assumptions and necessary conditions (stated in Section 1.3) [60].

### 7.6. Sensitivity analysis

**Fig 6:**
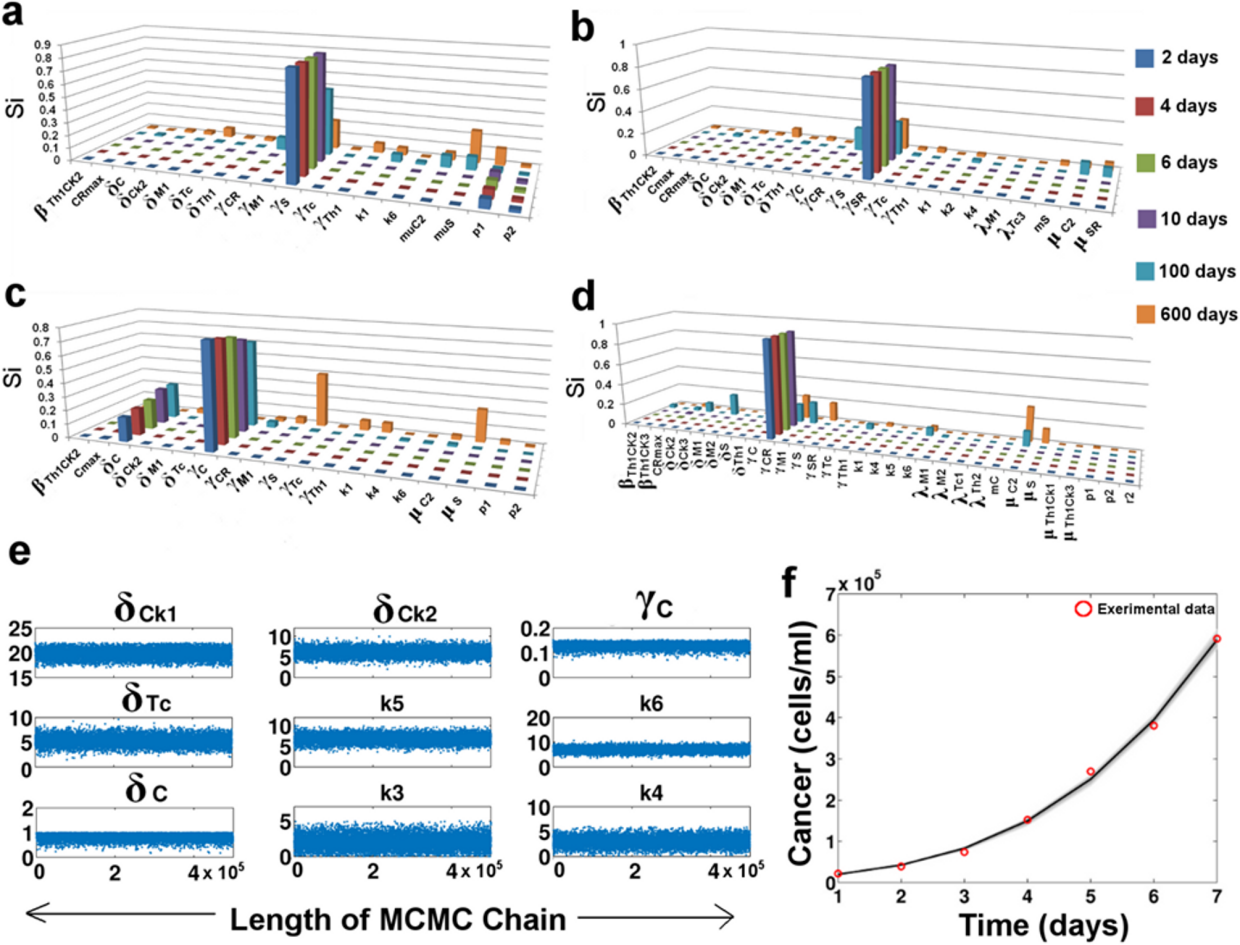
**Sensitivity analysis and parameter estimation:** (a-d) Sensitivity Analysis plots for S, S_R_, C and C_R_ respectively. The x-axis represents the parameters with p<0.05; (e) Trace Plot showing the best fitting parameter values as estimated by the MCMC algorithm; (f) Predictive plot of cancer proliferation using the estimated parameter values. The red circles represent the cancer cell proliferation values as obtained from Cell Counting experiments using Gastric Cancer cell line [29].

The sensitivity analysis of the model was performed by the extended Fourier Amplitude Sensitivity Test eFAST technique using a MATLAB based toolbox [61]. The sensitivity analysis was carried out using the whole set of parameters [k = 71]. 100 samples were chosen per search curve and resampling of the search curves was carried out 5 times [N_S_ = 100, N_R_ = 5]. Hence, the total number of model simulations N = (k+1)*Ns*N_R_ = 36000. The Sensitivity Indices (Si) of the parameters (p<0.05) for the variables governing the growth of the tumor sub-populations, *viz*. S, S_R_, C and C_R_ were estimated at different stages of the tumor development (Fig 6A–6D). Here it may be observed that at different time points that Si values of the parameters change, signifying the importance of the parameters in the different stages of the tumor development. The knowledge from this sensitivity analysis was used to determine the parameters that have a maximum effect on the tumor development.

### 7.7. Parameter estimation from Cancer cell line data

The model comprises of a total of 71 parameters. The values of 21 parameters of the model were curated from the existing literature. The unknown parameters were estimated (few were assumed within the biological feasible ranges) using the MATLAB based toolbox that employs the MCMC-DRAM algorithm for parameter estimation [62]. The time course experiment cytometric data for cancer cell proliferation obtained for 7 days in for Gastric cancer cell line (SGC7901) that was fitted for approximating the cancer cell behavior during the growth phase (Fig 6F) [29]. Parameters that were sensitive for the growth of the Cancer (C sub-population) cells were varied in biologically feasible ranges. The prior distribution was assumed to be normal and the MCMC simulation was carried out for 5 lakh iterations to ensure the convergence of the chain (Fig 6E). The estimated parameter values for the model have been listed in S1 Text.

### 7.8. Experimental data for model calibration

Apart from the parameters estimated using the MCMC method, the remaining unknown parameters governing the steady state behavior of the system were manually adjusted within the biologically feasible ranges for the calibration of the model so as to ensure that the simulation results corroborated with the various experimental observations. These parameter values (labelled as ’Expected’) used for the numerical simulation of the model have been enlisted in S1 Text. The experimental data used for this purpose were extracted from the available literature. For the validation of the growth kinetics of the resistant Cancer cells, time-course data of resistant cell lines were obtained from Breast cancer cell line MCF-7/TAX-resistant to Paclitaxel [31], Hepatocellular Carcinoma cell line SK-Hep1/CDDP3-resistant to Cisplatin [32], Colon Cancer cell lines SW-620-L-OHP and LoVo-L-OHP-resistant to Oxaliplatin [33]. The cytometric data obtained for the validation of the immune cell ratios were mostly obtained from Gastric Cancer, Ovarian Cancer and Osteosarcoma studies [34–39]. The data for the validation of cytokine expression were obtained from cases of Gastric and Breast Cancer studies [40–42]. These data used came from heterogeneous sources as none of the previously performed experiments were found to report the values of all cytometric data in a single experiment. Moreover, the use of data from the different Cancer studies ensures that the model is generic and mimics the average behavior observed in most Cancer studies. The use of data from both *in vitro* studies as well as data obtained from Cancer patients ensures the reliability of the model for its use in designing therapeutic control. In order to make the model specific for a single type of Cancer, one needs to simply obtain the cytometric data from a single experimental source and adjust the parameters accordingly.

### 7.9. Interior equilibria

To ensure positivity and existence of the interior equilibrium solutions, 36000 random parameter sets were generated (as mentioned in Section 7.6) within the biologically feasible ranges. Thereafter the model is simulated up to 800 days for each set of parameter. It was observed that each model simulation led to the positive interior equilibrium solution. Hence, we can state and prove the following Proposition.

**Proposition 2:** Positive interior equilibria exists for the set of equations Eqs 1–13 (Section 1.2).

Proof: The interior equilibrium points are the steady-state solutions of the Eqs 1–13 (Section 1.2) under the necessary conditions (Section 1.3) in the biologically feasible ranges of parameter values and initial conditions S1 Text.

### 7.10. Model initialization and numerical simulation

The tumor mass is formed by the sub-populations S, S_R_, C, C_R_. In our model, the S cell sub-population has been initialized to 1, while all the other tumor cell sub-populations have been considered as 0. The initial values of the remaining variables have been initialized based on cytometric data and cytokine expression values of healthy individuals, curated from the literature. The details have been provided in the S1 Text. The model was simulated numerically using the variable-step, variable order solver, ode15s, in MATLAB® 2017a platform.

## Supporting information

S1 Text Model parameters. The Supplementary Text contains the description of the parameters and state variables used for the model simulations.
(DOCX)

S1 Fig Treatment conditions under varying dose of Radiotherapy, Chemotherapy and Immunotherapy. The scatter plot depicts (a) the fold change of tumor population and (b) T_H1_/T_H2_ ratio under 1000 treatment combinations.
(TIF)
