# Supplementary material for: Exploring immuno-regulatory mechanisms in the tumor microenvironment: Model and design of protocols for cancer remission
Source: PLoS One. 2018 Sep 5;13(9):e0203030. doi: 10.1371/journal.pone.0203030 (PMC6124765; doi:10.1371/journal.pone.0203030)
Supplement: S1 Text — The Supplementary Text contains the description of the parameters and state variables used for the model simulations. (DOCX) [file pone.0203030.s001.docx]

**Supplementary Text S1**

**List of Parameters**

|  | **Parameter** | **Description** | **Units** | **Value** | **Reference^#^** |
| --- | --- | --- | --- | --- | --- |
|  | **β_M2_** | Rate of IL-10 production from M2 cells | ng cell^-1^day^-1^ | 1x10^-15^ | Expected |
|  | **β_Tc_** | Rate of IFN-γ production from Tc cells | ng cell^-1^day^-1^ | 1x10^-8^ | Expected |
|  | **β_Th1CK2_** | Rate of IFN-γ production from T_H1_ cells | ng cell^-1^day^-1^ | 1x10^-7^ | Expected |
|  | **β_Th1CK3_** | Rate of IL-2 production from T_H1_ cells | ng cell^-1^day^-1^ | 1 x10^-8^ | Expected |
|  | **β_Th2_** | Rate of IL-10 production from T_H2_ cells | ng cell^-1^day^-1^ | 1 x10^-9^ | Expected |
|  | **β_Treg_** | Rate of IL-10 production from Treg cells | ng cell^-1^day^-1^ | 1 x10^-10^ | Expected |
|  | **γ_C_** | Birth rate of Cancer cells | day^-1^ | 0.1282 | Estimated |
|  | **γ_CR_** | Birth rate of Cancer resistant cells | day^-1^ | 0.1282 | Expected |
|  | **γ_M1_** | Birth rate of M1 cells | day^-1^ | 0.7 | Expected |
|  | **γ_M2_** | Birth rate of M2 cells | day^-1^ | 0.01 | Expected |
|  | **γ_S_** | Birth rate of Stem cells | day^-1^ | 0.15 | Expected |
|  | **γ_Tc_** | Birth rate of Tc cells | day^-1^ | 1.0 | [[1](#_ENREF_1)] |
|  | **γ_Th1_** | Birth rate of T_H1_ cells | day^-1^ | 2.0 | [[2](#_ENREF_2)] |
|  | **γ_Th2_** | Birth rate of T_H2_ cells | day^-1^ | 2.0 | [[2](#_ENREF_2)] |
|  | **γ_Treg_** | Birth rate of Treg cells | day^-1^ | 0.3 | [[3](#_ENREF_3)] |
|  | **δ_C_** | Death rate of Cancer cells | day^-1^ | 0.8055 | Estimated |
|  | **δ_Ck1_** | Degradation rate of IL-10 | day^-1^ | 19.757 | Estimated |
|  | **δ_Ck2_** | Degradation rate of IFN-γ | day^-1^ | 6.1212 | Estimated |
|  | **δ_Ck3_** | Degradation rate of IL-2 | day^-1^ | 8.664339 | Calculated [[4](#_ENREF_4)] |
|  | **δ_CR_** | Death rate of Resistant Cancer cells | day^-1^ | 5.37 x10^-5^ | Estimated |
|  | **δ_M1_** | Death rate of M1 cells | day^-1^ | 1.02 | [[5](#_ENREF_5)] |
|  | **δ_M2_** | Death rate of M2 cells | day^-1^ | 0.05 | [[5](#_ENREF_5)] |
|  | **δ_S_** | Death rate of Stem cells | day^-1^ | 2 x10^-7^ | Expected |
|  | **δ_Tc_** | Death rate of Tc cells | day^-1^ | 5.2939 | Estimated |
|  | **δ_Th1_** | Death rate of T_H1_ cells | day^-1^ | 2.0 | [[6](#_ENREF_6)] |
|  | **δ_Th2_** | Death rate of T_H2_ cells | day^-1^ | 2.0 | Expected |
|  | **δ_Treg_** | Death rate of Treg cells | day^-1^ | 1.0 | [[7](#_ENREF_7)] |
|  | **λ_M1_** | Saturation constant for M1 activation | cells ml^-1^ | 1x10^8^ | Expected |
|  | **λ_M2_** | Saturation constant for M2 activation | cells ml^-1^ | 1 x10^6^ | Expected |
|  | **λ_Tc1_** | Saturation constant for Tc activation by cancer cells | cells ml^-1^ | 1 x10^5^ | Expected |
|  | **λ_Tc2_** | Saturation constant for Tc inhibition by stem cells | cells ml^-1^ | 5 x10^5^ | Expected |
|  | **λ_Tc3_** | Saturation constant for Tc inhibition by Treg cells | cells ml^-1^ | 5 x10^10^ | Expected |
|  | **λ_Tc4_** | Saturation constant for Tc activation by T_H1_ cells | cells ml^-1^ | 1 x10^5^ | Expected |
|  | **λ_Th1_** | Saturation constant for T_H1_ | cells ml^-1^ | 1 x10^5^ | Expected |
|  | **λ_Th2_** | Saturation constant for T_H2_ | cells ml^-1^ | 1 x10^5^ | Expected |
|  | **λ_Treg2_** | Saturation constant for Treg | cells ml^-1^ | 1 x10^7^ | Expected |
|  | **μ_C1_** | Rate of activation of C and C_R_ by IL-10 | day^-1^ | 0.75 | [[8](#_ENREF_8)] |
|  | **μ_C2_** | Rate of killing of C and C_R_ by IFN-γ | day^-1^ | 0.9 | [[9](#_ENREF_9)] |
|  | **μ_S_** | Rate of killing of S by IFN-gamma | day^-1^ | 0.17 | [[9](#_ENREF_9)] |
|  | **μ_SR_** | Rate of killing of S_R_ by IFN-gamma | day^-1^ | 0.18 | Expected |
|  | **μ_TcS_** | Rate of Tc killing by S and S_R_ cells | day^-1^ | 1 x10^-10^ | [[10](#_ENREF_10)] |
|  | **μ_TcTreg_** | Rate of Tc killing by Treg cells | day^-1^ | 1.5x10^-5^ | [[11](#_ENREF_11)] |
|  | **μ_Th1Ck1_** | Rate of T_H1_ killing by IL-10 | day^-1^ | 1 x10^-9^ | [[12](#_ENREF_12)] |
|  | **μ_Th1Ck3_** | Rate of T_H1_ activation by IL-2 | day^-1^ | 0.1245 | [[13](#_ENREF_13)] |
|  | **μ_TregCk1_** | Rate of Treg activation by IL-10 | day^-1^ | 1 x10^-7^ | Expected |
|  | **Cmax=K_tumor_/2** | Carrying capacity for Cancer cells **,** where K_tumor_=2 x10^10^ is the total Carrying capacity of non-stem tumor cells | cells ml^-1^ | 1 x10^10^ | [[14](#_ENREF_14)] |
|  | **CRmax=K_tumor_/2** | Carrying capacity for Resistant Cancer cells**,** where K_tumor_=2 x10^10^ is the total Carrying capacity of non-stem tumor cells | cells ml^-1^ | 1 x10^10^ | Expected |
|  | **k1** | Saturation constant for inhibition of S by IFN-γ | ng ml^-1^ | 10.0 | Expected |
|  | **k11** | Saturation constant for activation of Treg by IL-10 | ng ml^-1^ | 0.001 | Expected |
|  | **k2** | Saturation constant for inhibition of S_R_ by IFN-γ | ng ml^-1^ | 10.0 | Expected |
|  | **k3** | Saturation constant for activation of C by IL-10 | ng ml^-1^ | 2.0531 | Estimated |
|  | **k4** | Saturation constant for inhibition of C by IFN-γ | ng ml^-1^ | 3.02 | Estimated |
|  | **k5** | Saturation constant for activation of C_R_ by IL-10 | ng ml^-1^ | 6.7979 | Estimated |
|  | **k6** | Saturation constant for inhibition of C_R_ by IFN-γ | ng ml^-1^ | 6.9937 | Estimated |
|  | **k8** | Saturation constant for inhibition of T_H1_ by IL-10 | ng ml^-1^ | 0.01 | Expected |
|  | **k9** | Saturation constant for activation of T_H1_ by IL-2 | ng ml^-1^ | 0.001 | Expected |
|  | **ktc1** | Saturation constant for inhibition of S by Tc | cell ml^-1^ | 1 x10^9^ | Expected |
|  | **ktc2** | Saturation constant for inhibition of S_R_ by Tc | cell ml^-1^ | 1 x10^8^ | Expected |
|  | **ktc3** | Saturation constant for inhibition of C by Tc | cell ml^-1^ | 1 x10^9^ | Expected |
|  | **ktc4** | Saturation constant for inhibition of C_R_ by Tc | cell ml^-1^ | 1 x10^9^ | Expected |
|  | **m_C_** | Probability of Stem cell transformation into Stem resistant cells | - | 0.01 | Expected |
|  | **m_S_** | Probability of Cancer cell transformation into Cancer resistant cells | - | 4 x10^-7^ | [[14](#_ENREF_14)] |
|  | **p1** | Probability of Asymmetric differentiation of stem cells | - | 0.2 | [[14](#_ENREF_14)] |
|  | **p2** | Probability of Symmetric differentiation into two differentiated cancer cells | - | 0.05 | [[14](#_ENREF_14)] |
|  | **r1** | constant |  | 0.0001 | Expected |
|  | **r2** | constant |  | 1x x10^-5^ | Expected |
|  | **tck** | Rate of killing of tumor by Tc cells | day^-1^ | 0.1 | Expected |
|  | **µ_M1Ck2_** | Rate of M1 activation by IFN-γ | day^-1^ | 0.01 | Expected |
|  | **µ_M2Ck1_** | Rate of M2 activation by IL10 | day^-1^ | 0.01 | Expected |
|  | **k7** | Saturation constant for proliferation of M1 by IFN-γ | ng ml^-1^ | 0.2 | Expected |
|  | **k10** | Saturation constant for proliferation of M2 by IL10 | ng ml^-1^ | 0.01 | Expected |

**#** Estimated parameter values have been determined by the MCMC techniques using the time course experiment cytometric data for cancer cell proliferation for Gastric cancer cell line (SGC7901) (Refer *Methods* of Main Article) [15].

Expected parameter values are estimated by varying the parameters within the biologically feasible ranges found in various Literatures so as to determine its expected value to calibrate the model with experimental observations.

**List of Initial values**

| **Variable** | **Symbol** | **Initial Values** | **Reference** |
| --- | --- | --- | --- |
| Stem Cell | S | 1 | - |
| Stem Resistant Cell | S_R_ | 0 | - |
| Cancer Cell | C | 0 | - |
| Cancer Resistant Cell | C_R_ | 0 | - |
| Type-I Tumor Associated Macrophage | M1 | 85000 | Calculated [[5](#_ENREF_5)] |
| Type-II Tumor Associated Macrophage | M2 | 15000 | Calculated [[5](#_ENREF_5)] |
| Type-I Helper T Cell | T_H1_ | 71000 | [16] |
| Type-II Helper T Cell | T_H2_ | 12000 | [16] |
| Cytotoxic T Cell | Tc | 56000 | [16] |
| Regulatory T Cell | Treg | 8000 | [16] |
| Interleukin-10 | IL10 | 0.0085 | [17] |
| Interferon-γ | IFN-γ | 0.12 | [17] |
| Interleukin-2 | IL2 | 0.0094 | [17] |

**Reference**

1. Macallan DC, Asquith B, Irvine AJ, Wallace DL, Worth A, Ghattas H, et al. Measurement and modeling of human T cell kinetics. European journal of immunology. 2003;33(8):2316-26. doi: 10.1002/eji.200323763. PubMed PMID: 12884307.

2. Jansson A, Fagerlind M, Karlsson D, Nilsson P, Cooley M. In silico simulations suggest that Th-cell development is regulated by both selective and instructive mechanisms. Immunology and cell biology. 2006;84(2):218-26.

3. Vukmanovic-Stejic M, Zhang Y, Cook JE, Fletcher JM, McQuaid A, Masters JE, et al. Human CD4(+) CD25(hi) Foxp3(+) regulatory T cells are derived by rapid turnover of memory populations in vivo. Journal of Clinical Investigation. 2006;116(9):2423-33. doi: 10.1172/JCI28941. PubMed PMID: PMC1555646.

4. Robertson-Tessi M, El-Kareh A, Goriely A. A mathematical model of tumor-immune interactions. Journal of theoretical biology. 2012;294:56-73. doi: 10.1016/j.jtbi.2011.10.027. PubMed PMID: 22051568.

5. Italiani P, Boraschi D. From Monocytes to M1/M2 Macrophages: Phenotypical vs. Functional Differentiation. Frontiers in Immunology. 2014;5:514. doi: 10.3389/fimmu.2014.00514. PubMed PMID: PMC4201108.

6. Zhang X, Brunner T, Carter L, Dutton RW, Rogers P, Bradley L, et al. Unequal death in T helper cell (Th) 1 and Th2 effectors: Th1, but not Th2, effectors undergo rapid Fas/FasL-mediated apoptosis. Journal of Experimental Medicine. 1997;185(10):1837-49.

7. Xing S, Fu J, Zhang Z, Gao Y, Jiao Y, Kang F, et al. Increased turnover of FoxP3high regulatory T cells is associated with hyperactivation and disease progression of chronic HIV-1 infection. JAIDS Journal of Acquired Immune Deficiency Syndromes. 2010;54(5):455-62.

8. Enewold L, Mechanic LE, Bowman ED, Zheng Y-L, Yu Z, Trivers G, et al. Serum concentrations of cytokines and lung cancer survival in African Americans and Caucasians. Cancer Epidemiology and Prevention Biomarkers. 2009;18(1):215-22.

9. Briesemeister D, Sommermeyer D, Loddenkemper C, Loew R, Uckert W, Blankenstein T, et al. Tumor rejection by local interferon gamma induction in established tumors is associated with blood vessel destruction and necrosis. International journal of cancer. 2011;128(2):371-8.

10. Hof-Nahor I, Leshansky L, Shivtiel S, Eldor L, Aberdam D, Itskovitz-Eldor J, et al. Human mesenchymal stem cells shift CD8+ T cells towards a suppressive phenotype by inducing tolerogenic monocytes. Journal of Cell Science. 2012;125(19):4640.

11. McNally A, Hill GR, Sparwasser T, Thomas R, Steptoe RJ. CD4+ CD25+ regulatory T cells control CD8+ T-cell effector differentiation by modulating IL-2 homeostasis. Proceedings of the National Academy of Sciences. 2011;108(18):7529.

12. Sakamoto T, Saito H, Tatebe S, Tsujitani S, Ozaki M, Ito H, et al. Interleukin‐10 expression significantly correlates with minor CD8+ T‐cell infiltration and high microvessel density in patients with gastric cancer. International journal of cancer. 2006;118(8):1909-14.

13. Kirschner D, Panetta JC. Modeling immunotherapy of the tumor–immune interaction. Journal of mathematical biology. 1998;37(3):235-52.

14. Tomasetti C, Levy D. Role of symmetric and asymmetric division of stem cells in developing drug resistance. Proc Natl Acad Sci U S A. 2010;107(39):16766-71. doi: 10.1073/pnas.1007726107. PubMed PMID: 20826440; PubMed Central PMCID: PMC2947914.

15. Ning X, Sun S, Hong L, Liang J, Liu L, Han S, et al. Calcyclin-binding protein inhibits proliferation, tumorigenicity, and invasion of gastric cancer. Mol Cancer Res. 2007; 5(12): 1254-1262.

16. Frequencies of Cell Types in Human Peripheral Blood, www.stemmcell.com.

17. Kleiner, G., et al., Cytokine Levels in the Serum of Healthy Subjects. Mediators Inflamm. 2013; 2013: 434010.
