# Supplementary figures and images for: Exploring immuno-regulatory mechanisms in the tumor microenvironment: Model and design of protocols for cancer remission
Source: PLoS One. 2018 Sep 5;13(9):e0203030. doi: 10.1371/journal.pone.0203030 (PMC6124765; doi:10.1371/journal.pone.0203030)

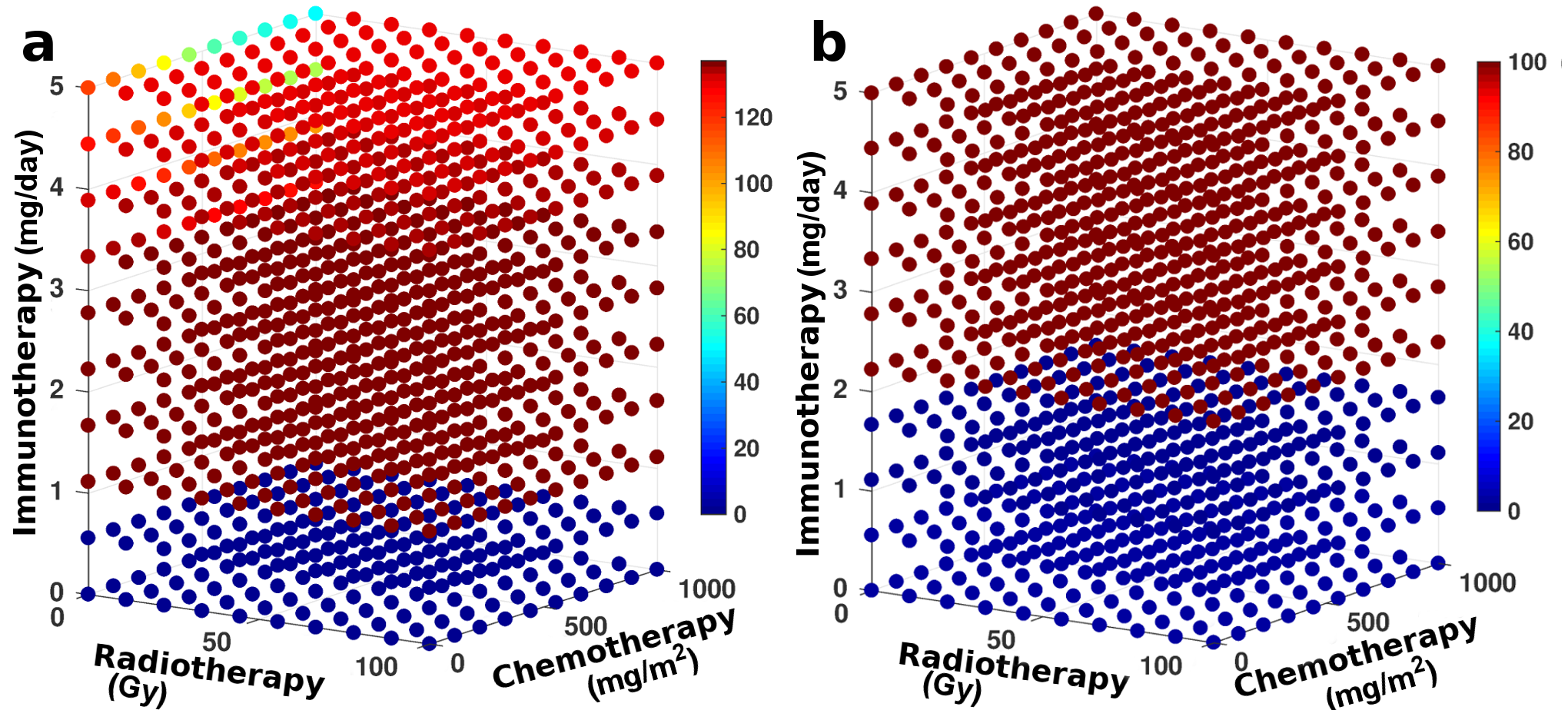

Supplement: S1 Fig — The scatter plot depicts (a) the fold change of tumor population and (b) TH1/TH2 ratio under 1000 treatment combinations. (TIF) [file pone.0203030.s002.tif]
